# Structural and functional basis of transcriptional regulation by TetR family protein CprB from *S. coelicolor* A3(2)

**DOI:** 10.1093/nar/gku587

**Published:** 2014-08-04

**Authors:** Hussain Bhukya, Ruchika Bhujbalrao, Aruna Bitra, Ruchi Anand

**Affiliations:** 1 Department of Chemistry, Indian Institute of Technology Bombay, Mumbai 400076, Maharashtra, India; 2 IITB-Monash Research Academy, Mumbai 400076, Maharashtra, India

## Abstract

Antibiotic production and resistance pathways in *Streptomyces* are dictated by the interplay of transcriptional regulatory proteins that trigger downstream responses via binding to small diffusible molecules. To decipher the mode of DNA binding and the associated allosteric mechanism in the sub-class of transcription factors that are induced by γ-butyrolactones, we present the crystal structure of CprB in complex with the consensus DNA element to a resolution of 3.25 Å. Binding of the DNA results in the restructuring of the dimeric interface of CprB, inducing a pendulum-like motion of the helix-turn-helix motif that inserts into the major groove. The crystal structure revealed that, CprB is bound to DNA as a dimer of dimers with the mode of binding being analogous to the broad spectrum multidrug transporter protein QacR from the antibiotic resistant strain *Staphylococcus aureus*. It was demonstrated that the CprB displays a cooperative mode of DNA binding, following a clamp and click model. Experiments performed on a subset of DNA sequences from *Streptomyces coelicolor* A3(2) suggest that CprB is most likely a pleiotropic regulator. Apart from serving as an autoregulator, it is potentially a part of a network of proteins that modulates the γ-butyrolactone synthesis and antibiotic regulation pathways in *S*. *coelicolor* A3(2).

## INTRODUCTION

*Streptomyces* species are well known for their wide variety of biologically active secondary metabolites and they also contribute to two-thirds of naturally occurring antibiotics (1,2). The synchronized behavior of these species in producing antibiotics and modulation of gene expression based on the variation in their cell-population density are governed by a cell-to-cell communicating system called quorum-sensing (QS). Cytoplasmically synthesized spectrum of small chemical signaling molecules, γ-butyrolactones (GBLs), diffuse freely through the cell membrane and participate in *Streptomycetes* QS mechanism as autoinducer molecules (3–5). When the concentration of GBLs reaches a stimulatory level both intra and extracellularly, the GBLs along with their cognate receptor proteins induce the regulon associated with antibiotic production, morphological differentiation and resistance biosynthetic pathways (5,6). ArpA (A-factor receptor protein A), a transcription factor from *S. griseus*, has been reported by Onaka *et al.* to be the first cognate receptor of GBL, A-factor (2-isocapryloyl-3-R-hydroxymethyl-γ-butyrolactone) (6–10). The 24-mer DNA consensus sequence (CS) for ArpA was identified through several rounds of polymerase chain reaction (PCR) amplification and immunoprecipitation experiments that were performed on a random pool of oligonucleotides (11). Using this 24-bp CS as a guide, Ohnishi *et al.* identified the promoter sequence of *adpA* to be the biologically relevant target DNA sequence for ArpA (12).

Apart from ArpA in *S. griseus*, the GBL receptor proteins include *S. lavendulae* FarA, an autoregulator of its own expression that controls blue pigment production with the help of butanolide IM-2 (13), *S. virginiae* BarA that controls virginiamycin biosynthesis (14,15) and TylP in *S. fradiae* that modulates tylosin biosynthesis (16). In *S. coelicolor* A3(2), a total of four proteins CprA, CprB (the *coelicolor* pigment regulator proteins) (17), ScbR (*S. coelicolor* QS receptors) and ScbR2, have been identified as GBL receptors (18–21). It was hypothesized in 1998 that both CprA and CprB are functional paralogs of the ArpA and activated the antibiotic biosynthesis and resistance genes in *S. coelicolor* A3(2) via the GBL QS pathway (19,21). Experiments performed with deletion mutants of *cprA* and *cprB* from *S. coelicolor* A3(2) exhibited acute reduction in antibiotic production and altered the sporulation time (19). However, till date, small molecules responsible for triggering transcriptional activity of CprA and CprB and their target DNA sequence remain obscure. Instead ScbR was identified as the functional homolog of ArpA and it was shown to follow an interaction and activation mechanism similar to that observed for the ArpA-*adpA* system (22). Similar to CprA and CprB, ScbR also regulates antibiotic production in *S*. *coelicolor* A3(2) and in particular it has been reported to control the metabolites of the cryptic type I polyketide synthase gene cluster (23). Recently ScbR2, a pseudo-GBL receptor, has been shown to bind indigenous antibiotics produced in *S*. *coelicolor* A3(2) and thereby regulate both antibiotic and GBL synthesis pathways (18,24). However, whether these GBL receptors in *S*. *coelicolor* A3(2) function independently or as a part of a regulatory network that connects them is not well understood.

In order to shed light on the domain organization of the GBL receptor class of transcription regulators, Natsume *et al.* solved the crystal structure of the apo form of CprB (21). The structure revealed that it belongs to TetR (tetracycline repressor) superfamily of transcriptional regulators (TetR-FTRs) comprising a DNA binding domain (DBD) and a ligand-binding domain (LBD) at the N- and C-terminus of the protein, respectively. The DBD recognizes and interacts with the cognate operator sequence (OS) through the helix-turn-helix (HTH) motif, whereas the LBD regulates the DNA binding activity by interacting with its cognate inducer molecule (21,25). TetR-FTRs exhibit a high degree of conservation in the amino acid sequence of the DBD, conversely, the LBD is divergent across this family. This suggests that the LBD of TetR-FTRs responds to diverse inducer molecules that regulate different pathways involved in various biological functions (21,25–26).

Till date, seven protein–DNA complex structures from the TetR-FTRs have been reported, which are *Escherichia coli* TetR (27), *Staphylococcus aureus* QacR (28), *Pseudomonas aeruginosa* DesT (29), *Corynebacterium glutamicum* CgmR (30), *Streptomyces antibioticus* SimR (31), *E. coli* SlmA (32) and *Mycobacterium smegmatis* Ms6564 (33). Four of these proteins, TetR, QacR, CgmR and SimR assist in conferring resistance to certain antibiotics or toxins that the host organism is exposed to. For example, TetR is responsible for the efflux of the tetracycline-magnesium ion (Mg^2+^) complex (34–38), SimR regulates the export of simocyclinone (39) and QacR binds to a broad spectrum of quaternary ammonium cationic compounds, regulating the transcription of the multidrug transporter, *qacA* (40). Similarly, CgmR binds to antibiotics like ethidium and methylene blue; it has been proposed to be a multidrug resistance regulator (30). On the other hand, DesT regulates the genes that maintain the ratio of unsaturated:saturated fatty acid levels in the organism (41), whereas SlmA is involved in the nucleoid occlusion and prevents cytokinetic Z-ring formation during cell division (42,43). Unlike others, Ms6564 is a master regulator of genes that are responsible for DNA damage/repair mechanism (44).

There is a paucity of structural information in the GBL family of proteins and there are no available structures of the GBL receptor (sub-class of TetR-FTRs) in the DNA-bound form. Hence, here we illustrate the mechanism of DNA binding in GBL receptor family using CprB from *S. coelicolor* A3(2) as a model system. The crystal structure of CprB in complex with the CS was determined to a resolution of 3.25 Å. The structure of CprB–CS complex was compared with other structurally characterized TetR-FTRs and a model of the operator action for CprB was proposed. In order to identify a subset of the DNA elements that CprB targets, a genome-wide search in conjunction with electrophoretic mobility shift assays (EMSAs) was employed. The stoichiometry, affinity and mode of binding of CprB with DNA sequences were established using isothermal calorimetric (ITC) experiments. To recognize the role of key amino acids in DNA binding, various site-directed mutational studies were performed in the HTH motif of the DBD in CprB.

## MATERIALS AND METHODS

### Overexpression and purification of CprB

The *cprB* gene from the *S. coelicolor* A3(2), which encodes 215 amino acid protein was expressed *in vivo* in the *E. coli* system. The vector, pET26b(+) containing *cprB* (obtained from Ryo Natsume, Japan Biological Informatics Consortium, Tokyo, Japan) was introduced into *E. coli* expression cells, BL21(DE3)pLysS. An overnight culture of 5-ml Luria-Bertani (LB) medium containing 35 μg/ml kanamycin and 30 μg/ml chloramphenicol was inoculated into 1 l of LB medium, which also had the same concentration of antibiotics. Consequently, the culture was grown at 37°C with constant shaking at 250 rpm until the OD_600_ reached 0.4–0.5. CprB expression was induced by adding isopropyl-β-thiogalactopyranoside (IPTG) to a final concentration of 1 mM (45) and grown for 3 h at 37°C. The culture was then cooled and grown at 25°C for 3 h. The bacterial cells grown were harvested by centrifugation at 4000 rpm for 20 min and then the cell pellet was re-suspended in 15–20 ml buffer A (50 mM phosphate buffer at pH 7) (45) and are homogenized by a probe sonicator (Vibra-cell; SONICS, CT, USA). All the subsequent protein purification steps were carried out at 4°C. Cell debris was removed by high speed centrifugation at 20 000 rpm for 50 min. The supernatant was then mixed with SP Sepharose beads (GE Healthcare, WI, USA), which were pre-equilibrated in buffer A (45) and gently stirred on a gel rocker for 1 h. The beads were then separated by centrifugation and transferred into a column, followed by a 6-h wash using buffer A (∼100 ml). Protein elution was performed with a linear gradient of NaCl (100–400 mM) in buffer A. The eluted fractions of pure protein were desalted using an Econo-Pac 10DG (Bio-Rad, CA, USA) column pre-equilibrated with buffer B (50 mM phosphate buffer at pH 7 and 150 mM NaCl). The desalted fractions of CprB were rebound to the beads and eluted with 1 M NaCl in buffer A. Finally, the protein was desalted with buffer B and stored at 4°C. Protein concentrations were quantified in a spectrophotometer by measuring the absorbance at 280 nm. The purity of the protein was verified by running an sodium dodecyl sulphate-polyacrylamide gel electrophoresis (SDS-PAGE) analysis with 15% polyacrylamide gel followed by Coomassie Blue (HiMedia, Mumbai, India) staining. All the mutants (C159S, Y47A, K43A, T31A, S33A and a double mutant, S33A and K43A) were overexpressed and purified by performing the same protocol.

### Synthesis of oligonucleotides

All DNA oligonucleotides sequences (Supplementary Table S1) used in EMSA studies were synthesized using MerMade4 (Bioautomation, Plano, Texas, USA) automated synthesizer at 1 μmol scale with suitable controlled pore glass (Proligo Reagents, Hamburg, Germany) beads as a 3’ solid support. The synthesized oligonucleotides were deprotected and purified by denaturing PAGE (20%, 7 M urea) employing standard protocols. Quantification of all the oligonucleotides listed in Supplementary Table S1 was done at 260 nm using an ultraviolet-visible spectrophotometer (GeneQuant 1300; GE Healthcare, WI, USA) with the appropriate molar extinction coefficients (*ϵ*). The complimentary strands (1:1 ratio in concentration) were annealed by heating at 95°C for 5 min [in a buffer containing 5 mM Tris–HCl, pH 7.5, 15 mM NaCl and 0.1 mM ethylenediaminetetraacetic acid (EDTA), pH 8.0] and allowed to cool slowly to room temperature, after which, they were stored at –20°C.

### Radiolabeling of oligonucleotides

The 5’-end of oligonucleotide were labeled to carry out EMSA studies. A 10 pmol of unlabeled DNA was mixed with 1× T4 polynucleotide kinase (PNK) buffer [50 mM Tris–HCl (pH 7.6), 10 mM MgCl_2_, 5 mM DTT and 0.1 mM spermidine]. The T4 PNK enzyme (Fermentas, Pittsburgh, PA, USA), 5 U and [γ-^32^P] ATP (3300 Ci/mmol) (BRIT, Hyderabad, India) were further added in a total volume of 10 μl. After incubating the reaction mixture at 37°C for 1 h, the enzyme was deactivated by heating the reaction mixture to 70°C for 3 min. The end labeled product was then isolated from the reaction mixture using the QIAquick nucleotide removal kit (Qiagen GmbH, Hilden Germany) protocol provided by Qiagen.

### Electrophoretic mobility shift assay

CprB–DNA binding assays were carried out using 5’-end radiolabeled oligonucleotides. Approximately, 1 nM of annealed DNA (∼5000 cpm) was incubated with a two-fold serially diluted protein (starting from 6 μM to 23 nM) at 20°C for 30 min in a buffer containing 10 mM Tris–HCl (pH 7.8), 50 mM KCl, 1 mM EDTA (Ethylenediaminetetraacetic acid.), 1 mM dithiothreitol (Sigma) and 5% (vol/vol) glycerol. In addition, the buffer also contains 10 mg hemoglobin (Sigma) and 2–3 μg of Poly(dI-dC)·Poly(dI-dC) (Sigma) in a total volume of 20–40 ml (46). After incubation, the samples were run on 6% non-denaturing polyacrylamide gel with 1× Tris-Borate-EDTA (TBE) as a running buffer (89 mM of each Tris and boric acid and 2 mM of EDTA, pH 8.3) at 4°C and 100 V for 1 h. EMSA results were collected and analyzed on Storm625 (GE Healthcare, WI, USA) and autoradiograms were generated using the ImageQuantTL software provided by GE Healthcare.

### Site-directed mutagenesis

The *cprB* gene cloned vector, pET26b(+) was used as a template for site-directed mutagenesis studies. The forward primers used for S33A, K43A, Y47A and C159S mutants are 5′-TCGACGACCCTGGCCGAGATAGTAGCC-3′, 5′-GCCGGGGTCACCGCGGGCGCCCTGTAC-3′, 5′-AAGGGCGCCCTGGCCTTCCACTTCGCG-3′ and 5′-CACACCCTCGTCTCCTCCGTCGTCGGC-3′, respectively, and the reverse primers are 5′-GGCTACTATCTCGGCCAGGGTCGTCGA-3′, 5′-GTACAGGGCGCCCGCGGTGACCCCGGC-3′, 5′-CGCGAAGTGGAAGGCCAGGGCGCCCTT-3′ and 5′-GCCGACGACGGAGGAGACGAGGGTGTG-3′, respectively. The reaction mixture contained 1× KapaHiFi buffer, 0.2 mM deoxyribonucleotide triphosphate (all the PCR chemicals were supplied by Genetix Biotech Asia Pvt. Ltd., Mumbai, India), 1.6 μM of each of the primers, 1 ng/μl of template DNA and 1 U of KapaHiFi polymerase in 50 μl reaction mixture. To the PCR product, 1 μl Dpn1 (20 000 units/ml) was added and incubated at 37°C for 90 min. The Dpn1-digested PCR product was transformed into *E. coli* competent cells, DH5α (for plasmid isolation) and subsequently introduced into BL21(DE3)pLysS cells for protein production as mentioned above for native CprB.

### Co-crystallization of CprB with synthesized oligonucleotides

Purified native CprB, 6 mg/ml was mixed with annealed 22-mer CS in the ratio of 1:1.2 (dimer of CprB:CS) and incubated at 20°C for 30 min. Co-crystallization trials of CprB–CS were performed with crystallization screens; Natrix HR116 and Natrix2 HR117 from Hampton Research, CA, USA employing hanging-drop vapor diffusion method. Each drop contained 2.0 μl of CprB–CS and 1.5 μl of 200-μl well solution. Crystallization plates were stored at 22°C and the crystals were obtained in the condition with 0.2 M KCl, 0.02 M MgCl_2_·6H_2_O, 0.05 M Tris–HCl pH 7.5 and 10% polyethylene glycol 4000 after 2 weeks. The CprB–CS crystals were cryoprotected with 20% ethylene glycol.

### Data collection and refinement

All the crystals were flash cooled using liquid nitrogen and mounted onto the goniostat at the BM-14, European synchrotron radiation facility (ESRF, Grenoble, France). Data were collected for 8 s of exposure at every 1° oscillation on MAR CCD detector. The resultant data were integrated using iMOSFLM (47), and subsequently, scaled by SCALA (48) program from the CCP4i suite. The data from CprB–CS complex were collected to 3.25 Å resolution. The coordinates of apo CprB (PDB entry: 1UI5) were used for molecular replacement and the initial search was performed using Auto-Rikshaw (49). The asymmetric unit contains two homodimers of CprB and a double-stranded CS DNA. An idealized B-form of CS DNA was manually fit into the electron density using COOT (50), since the starting two DNA bases (dA and dC) of chain E in the complex were disordered, they were not included in the refinement. The structure was then refined using Crystallography and NMR System (CNS) (51) and REFMAC5 (52). Figures were rendered using PyMol (53) and the helical parameters of CS were calculated using Web-3DNA (54).

### Thermodynamics of binding (CprB–DNA)

Calorimetric experiments were carried out using MicroCal iTC200 (GE Healthcare, WI, USA). CprB and DNA samples [CS/operator of CprB (OPB)] were diluted in buffer B and centrifuged at 6000 rpm for 5 min. To nullify the heat of dilution, DNA was titrated against the buffer B and subtracted from the raw data prior to fitting. In both ITC experiments, the sample cell containing 20 μM CprB was titrated with 120 μM annealed DNA. The volume of the titrant added at each injection into the sample cell was 1.5 μl for 4 s. The time interval between the successive injections is 120 s. The temperature of the calorimeter cells (sample and reference) was maintained at 25°C. The data obtained for CprB–CS complex were fit using one set of sites models, whereas the CprB–OPB data were fit using two sets of sites model in Origin 7 (provided with the instrument).

## RESULTS

### Structural characterization of DNA-bound CprB and comparison with its apo form

CprB consists of 10 α-helices, among which, three of the N-terminal α-helices α1, α2 and α3 form the core DBD, with spacer helix α2 (residues 33–39) and recognition helix α3 (residues 43–49) constituting the signature HTH motif, commonly present among transcription regulators. The remaining seven helices, α4–α10, constitute the dimerization and the LBDs with α4 serving as a connector helix that transmits the information between the various states of the protein. The asymmetric unit of the CprB–CS complex consists of two CprB dimers and a double-stranded DNA. The data reduction and refinement statistics are listed in Table 1. The DNA sequence used for complexation was semi-palindromic (5′-ACATACGGGAC*GCCCCGTTTAT-3′, where the asterisk represents the dyad axis) and has been previously shown by Sugiyama *et al.* to bind ArpA as well as CprB (46).

**Table 1.** Crystallographic data processing and refinement statistics

Space group	*P*3_2_
Unit cell dimensions (Å)	*a* = 149.06, *b* = 149.06, *c* = 69.07
Resolution (Å)	60.9 − 3.25 (3.43 − 3.25)^a^
Wavelength (Å)	0.98
Total no. of reflections	123 664 (182 65)^a^
No. of reflections in working set	26 013
No. of reflections in test set	1080
Total no. of unique reflections	27 093 (3988)^a^
Redundancy	4.6 (4.6)^a^
Completeness	100 (100)^a^
*R_merge_* (%)	11.5 (29.3)^a^
*I*/*σ*	8.4 (3.1)^a^
*R*_work_/*R*_free_	21.83/29.42
B-factor (Å^2^)	
Mean B-value for overall structure	49.47
Mean B-value for protein	59.63
Mean B-value for DNA	39.32
Ramachandran favored (%)	89.08
Ramachandran allowed (%)	8.58
Ramachandran outliers (%)	2.34
RMSD bond distance (Å)	0.01
RMSD bond angle (°)	1.82
PDB entry	4PXI

^a^The figures in brackets signify the values for highest resolution shell. *R*_merge_=Σ*_hkl_* Σ*_i_* |*I_i_*(*hkl*) − {*I*(*hkl*)}|/ Σ*_hkl_* Σ*_i_I_i_*(*hkl*), where *I_i_*(*hkl*) is the *i*th observation of reflection *hkl* and {*I*(*hkl*)} is the weighted average intensity for all observations *i* of reflection *hkl*. The R-factors *R*_work_ and *R*_free_ are calculated as follows: *R*= Σ(|*F*_obs_ − *F*_calc_|)/Σ|*F*_obs_| × 100, where *F*_obs_ and *F*_calc_ are the observed and calculated structure factor amplitudes, respectively.

**Figure 1.**
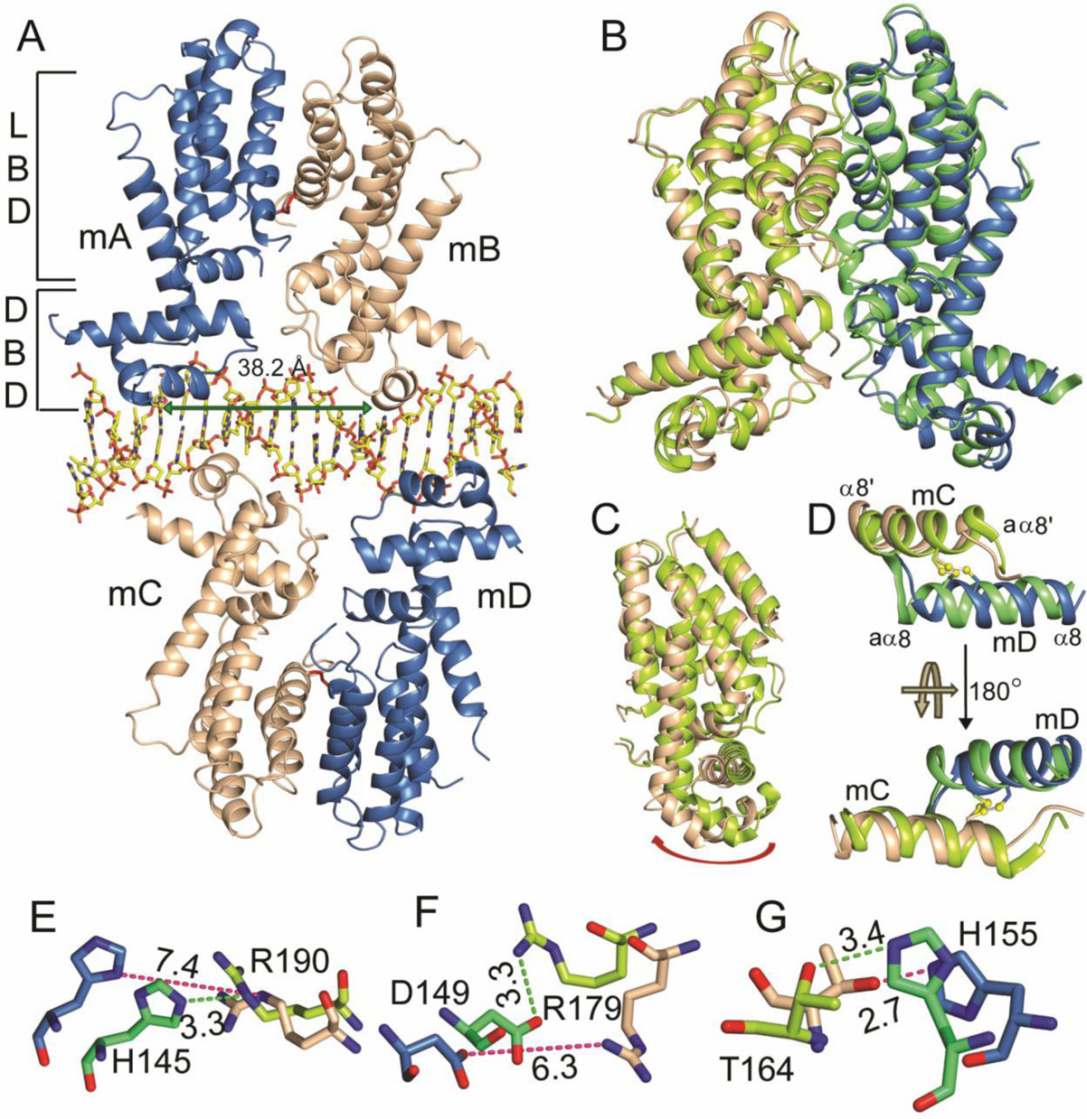
The X-ray crystal structure of CprB in complex with CS DNA. (**A**) The overall view of the complex structure. The two homodimers of CprB bound to double-stranded CS are represented in a cartoon model and the CS DNA in stick model. The monomers are labeled as mA, mB, mC and mD. The green arrow shows the distance between two recognition helices, α3 from each monomer of the homodimer. (**B**) Conformational changes upon DNA binding: superposition of the DNA-bound and apo forms of CprB homodimer. The monomers of apo form are represented in green and monomers of DNA-bound form are represented in blue and wheat. The superposition was performed by using the LBD domain as a reference frame. (**C**) Highlights the pendulum-like shift in the DBD domain of the complex form of CprB. (**D**) Zoomed in view near the disulfide bond region. Intermonomeric disulfide bond is highlighted in ball and stick representation. (**E**–**G**) Representative interactions that are restructured along the dimerization interface upon DNA binding. Color coding in (D–F) is same as mentioned in (B) and (C). All distances are in Å.

In contrast to the apo form of the protein, which is a dimeric unit, the CprB–CS complex was found to be a dimer of dimers. Both the dimeric units are bound at opposite sides of the 22-bp CS and there are no interactions between the two dimers. The center-to-center distance between the two monomers of a homodimer is 38.2 Å (measured from amide nitrogen atom of G44 from both the recognition helices α3 of homodimer), as shown in Figure 1A. The CprB consists of 215 amino acids; however, due to the weak and/or no electron density observed for the residues 1–4, 113, 114, 165–175 and 212–215 in monomer A, 1–4, 166–169 and 213–215 in monomer B, 1–4, 168–174 and 213–215 in monomer C and 1–7, 77–79, 118, 119, 167–173 and 213–215 in monomer D, they were not included in the final refined structure. Similar to the apo form of the CprB, within a dimeric unit of the CprB–CS complex, the two monomers possess a pseudo two-fold symmetry axis. The monomers of a homodimer are covalently connected via a disulfide linkage between cysteine residues at position 159. In the apo form of CprB, the nature of this disulfide bond is LH (left handed) spiral with strain energy of 4.18 kcal/mol. There is no conformational change in the disulfide bond of CprB upon binding to the CS; however, there is an increase in strain energy upon binding (5.12 kcal/mol for monomers A and B and 6.5 kcal/mol for monomers C and D; analysis of disulfide bond was done using web server, http://149.171.101.136/python/disulfideanalysis/search.html). In general, the LH spiral disulfide bonds are known to confer structural stability (55–57). Similar scenario was also observed in another TetR-FTR protein, SbtR (*Thermus thermophilus* HB8) where the disulfide bond possess a LH spiral geometry and partakes in stabilizing the dimeric unit (57). To shed light on the role played by the disulfide bond in CprB, cysteine 159 was mutated to serine. The mutant form (C159S) could not be expressed in appreciable amounts to conduct further experiments. The dramatically reduced expression level indicates that the disulfide bond is important for structural stability of the protein. Comparative analysis of the CprB–CS complex and the apo form of CprB was performed using LBD domain as a reference frame (Figure 1B). Binding of the CS to CprB induces a general pendulum-like movement in the overall protein structure as shown in Figure 1C. A twist in the dimeric interface results in a coordinated motion of the DNA binding HTH motif about the connector helix α4. This motion facilitates a snug fit of helix α3 into the major groove of the CS, thereby aiding DNA binding event. A minimal shift of 1 Å in the position of the disulfide bond was observed (as shown in Figure 1D) for the dimeric unit formed by monomers C and D, thereby highlighting the fact that it acts like a tether between the two subunits. It is possible that the two CprB dimers utilize the disulfide bond as a fulcrum to rotate between conformational states.

### Interactions in the dimeric interface

The dimeric interface of CprB is stabilized by an extensive network of hydrogen bonding and hydrophobic interactions between the α-helices α8 and α9 from both the subunits. Due to pivot-like motions induced upon DNA binding, there are several contacts that are readjusted along helices α8 and α9 at the dimeric interface. A total of 10 hydrogen bonds are disrupted and approximately nine different electrostatic connections are created. For example, the interactions disrupted are the side chain nitrogen atom NE2/ND1 of H145 in monomer A to the nitrogen atom NE from the side chain of R190 in monomer B, which was 3.3 Å in the apo structure and is around 7.4 Å in the DNA-bound form, shown in Figure 1E. Similarly, the distance between the side chain of the oxygen atom OD2 of D149 of monomer A to the side chain nitrogen atom NH1 of R179 of monomer B changes from 3.3 to 6.3 Å in the two forms, as represented in Figure 1F. Furthermore, the new hydrogen bonding interactions that are created, for instance, are the side chain nitrogen atom NE2 of H155, which was 3.5 Å away from the OG1 oxygen atom of T164 in the apo structure and now forms a hydrogen bond (2.7 Å) in the DNA-bound form shown in Figure 1G. The complete details of interactions that are restructured along the dimeric interface are tabulated in Supplementary Tables S2 and S3. There are several hydrophobic interactions that are also altered as a result of this fluidity of the interface region. The calculation of the solvent accessible surface area of the apo and the DNA-bound forms of the protein has values of around 18267.9 and 19838.1 Å^2^ [calculated using CNS (51)], respectively. The ligand pocket volumes also change from ∼635 Å^3^ in the apo form to ∼750 Å^3^ in the DNA-bound form, indicating that the operator-bound form of the dimer is a more open structure with an increased accessibility to the ligand-binding pocket. Therefore, it appears that binding of DNA to CprB primes it to accept the regulatory small molecule that controls its transcriptional activity.

### CprB–CS interactions

**Figure 2.**
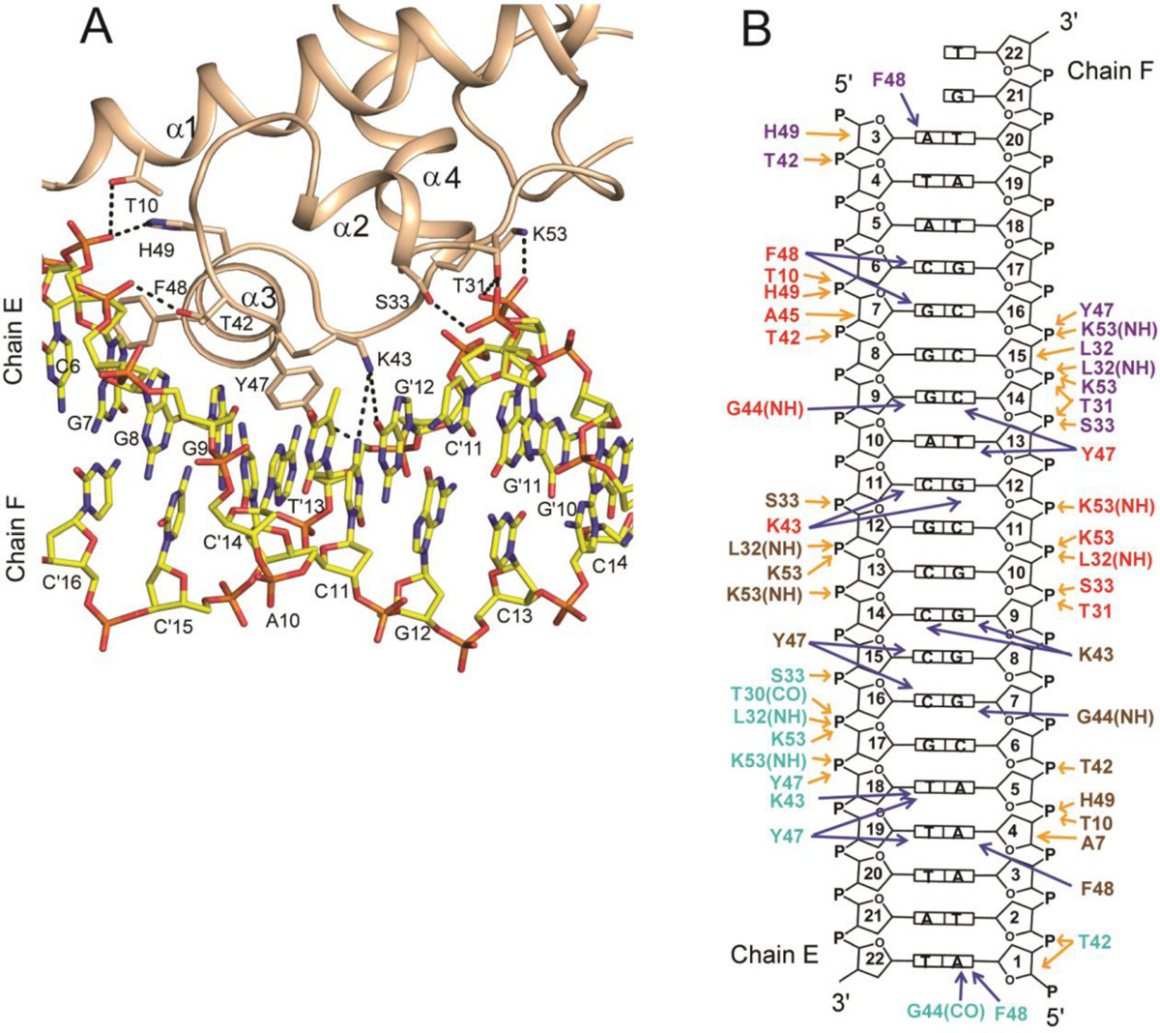
Detailed intermolecular interactions between CprB and CS. (**A**) The interactions of HTH with the bases in the major groove and phosphate backbone of CS are shown. The hydrogen bonding interactions are pointed as dashed lines. The DNA bases indicated using prime (A', T', G' and C') correspond to chain F of CS in CprB–CS complex (PDB entry: 4PXI) and the others are from the complementary strand (chain E). (**B**) Schematic representation of protein interaction with CS. Residues colored in purple, brown, red and cyan are from subunits A, B, C and D, respectively of the CprB–CS complex. The blue and orange colored arrows indicate the base and backbone interacting residues of CprB respectively with CS.

To facilitate the interaction of the HTH motif with the CS, the spacer helix α2 orients such that it results in widening of the groove to ∼13 Å (ideal B-form has 11.7 Å). This allows the helix α3 to insert and make protein DNA contacts to achieve specificity of binding via an induced fit mechanism. This effect of the deformation of the DNA is transmitted along the length of the chain causing the adjacent major groove, not interacting with the protein, to be shrunk by ∼10 Å (Supplementary Table S4). The average of all the roll and twist angles in the DNA are around 0.9° and 35.5°, respectively, yielding a global bend of ∼3.5° in the DNA as compared to the standard B-form. The CprB–CS complex is stabilized by a total of around 35 phosphate backbone and 20 direct base contacts, shown in Figure 2A and B. The direct base contacts in the monomers are mostly through the residues K43, G44, Y47 and F48, whereas residues H49, Y47, T42, S33, T31 and K53 interact via the phosphate backbone. Residues Y47, K43, G44 and F48 from α3 of the HTH motif are tightly docked into the major groove of the CS DNA and are the four major amino acids that partake in base contacts. The hydroxyl moiety of residue Y47 forms a hydrogen bond with the phosphate backbone of all the monomers and the T-shape stacking of the phenyl ring occurs with the bases from the DNA major groove. In several instances, due to the heterogeneity of the DNA sequence, these residues in each of the four monomers encounter different bases, creating a diverse environment. For example, K43 in monomer B, C and D forms hydrogen bond contacts with the bases dG’9, dG’12, dC11, dC14 and dT18; however, K43 in monomer A is disordered. Overall, in this scenario, both the hydrophobic region and the positively charged side chain of the K43 interact with various bases of the DNA via non-covalent interactions. Similarly, in the case of F48 in all the four monomers, a difference in the binding was observed. The F48 in monomers A and C stacks with guanine bases, dA3 and dC6, respectively. Whereas the F48 in monomers B and D stacks with dA’4, and dA’1, respectively (Figure 2A and B).

### Identification of the cognate DNA for CprB

**Figure 3.**
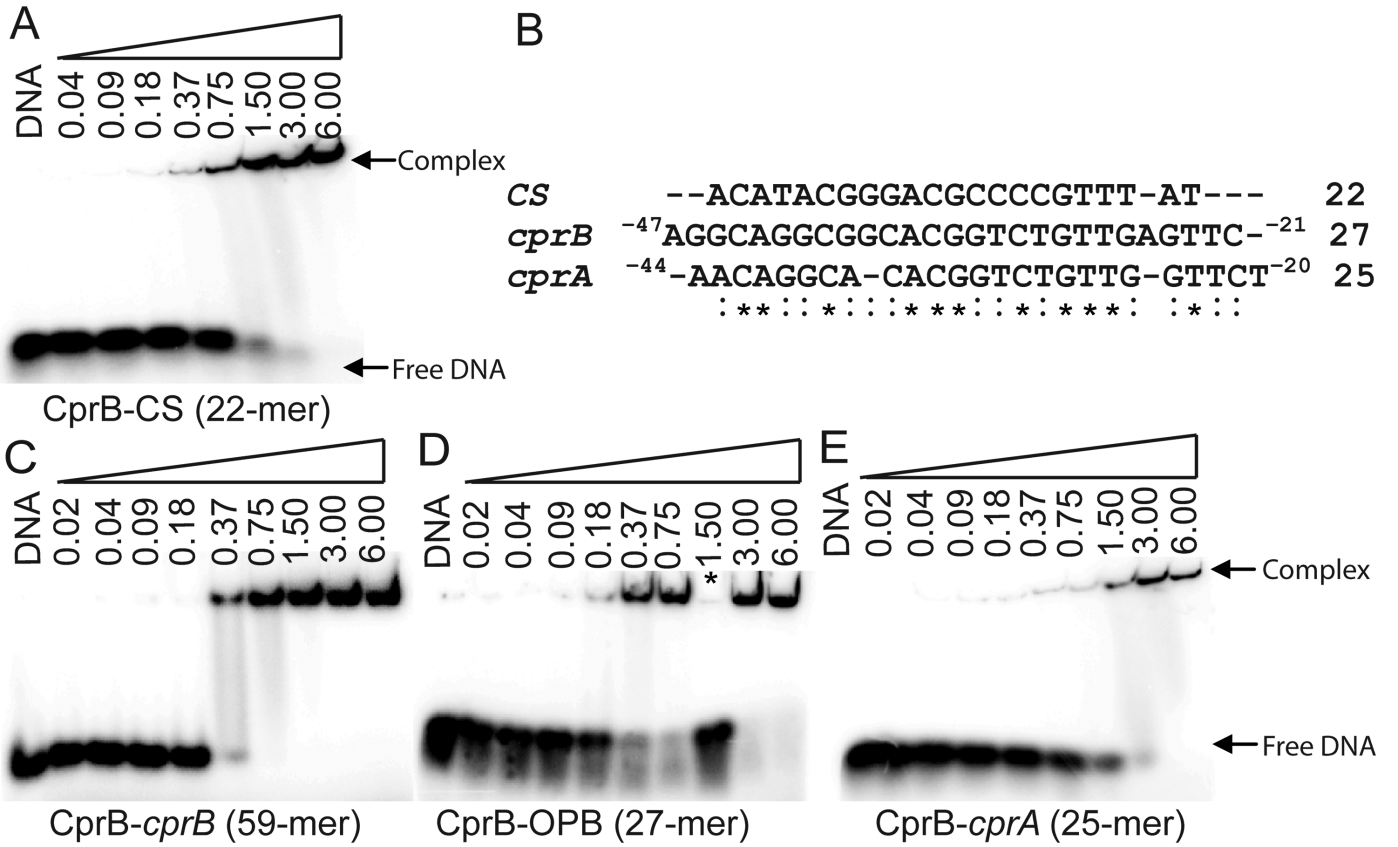
Gel shift assays carried out using purified CprB and radiolabeled DNA. (**A**) Result of CprB with 22-mer CS complex formation. (**B**) Multiple sequence alignment of the upstream regions of *cprA* (25-bp) and *cprB* (27-bp) with CS; asterisk indicates the bases conserved in all three sequences and colon indicates the bases conserved in any two sequences aligned. (**C**–**E**) The EMSA results showing the CprB–DNA complex formation of purified CprB with longer stretch, 59-mer of upstream sequence (−58 to 0) from *cprB*-ATG, shorter sequence, 27-mer (−47 to −21) OPB and the upstream sequence, 25-mer (−44 to −20) of *cprA*-ATG, respectively. The final concentration of CprB is indicated above each lane. The band corresponding to CprB–DNA (complex) and free DNA are indicated. Lane highlighted using asterisk in (D) has excess of cold OPB DNA along with the reaction mixture. All the concentrations are mentioned in micromolar.

**Figure 4.**
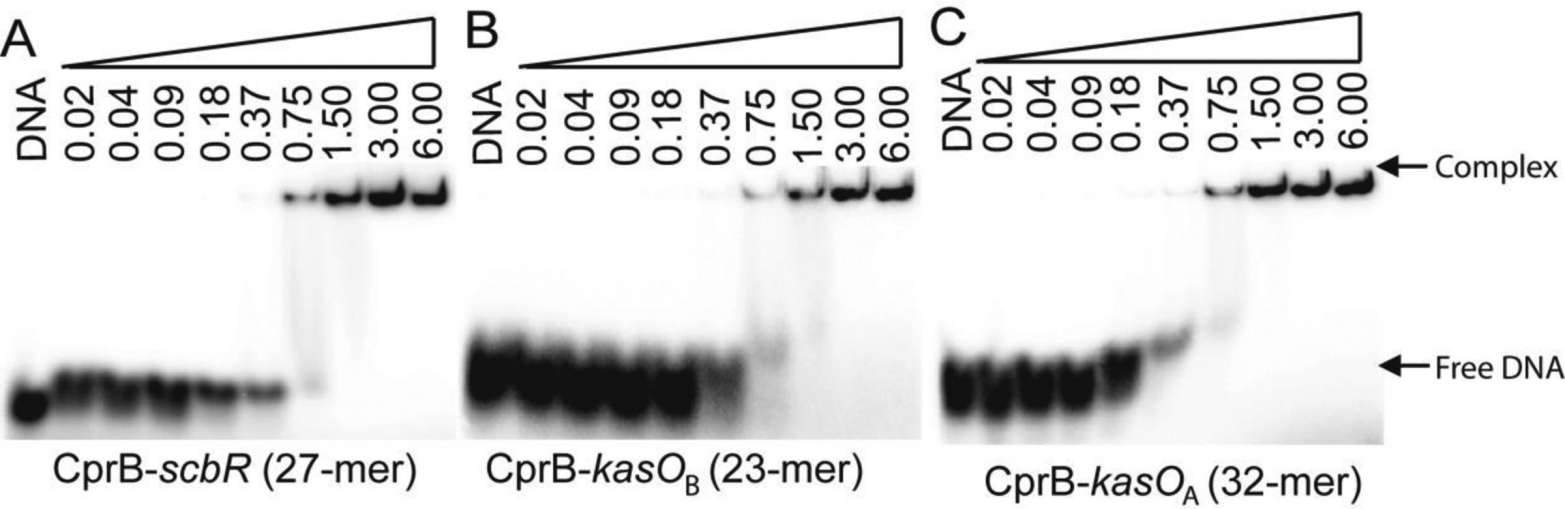
Results of DNA retardation assay performed with CprB and the ScbR recognition sequences. (**A**) The promoter of *scbR* (27-mer). (**B** and **C**) Two sites (*kasO*_A_, 32-mer and *kasO*_B_, 23-mer) in the promoter of the cryptic type I polyketide synthase gene cluster. All the concentrations are mentioned in micromolar.

Sugiyama *et al.* in 1998 have demonstrated that CprB binds to a 22-bp imperfect palindromic CS that has been established to bind the ArpA of *S. griseus* (46). We employed EMSA studies to confirm and estimate the DNA binding affinity of CprB toward the CS and the values were found to be in the range of 350–400 nM shown in Figure 3A. Although this DNA sequence was instrumental in understanding the conformational changes via the CprB–CS complex structure, the identity of the cognate DNA that CprB regulates in *S. coelicolor* A3(2) is not known till date. Therefore, to identify the biologically relevant DNA sequence recognized by CprB in its parent organism, we performed a genome-wide search of *S. coelicolor* A3(2) by using CS as the search string. The search identified the upstream regions of *cprA*-ATG and *cprB*-ATG to be the most similar in sequence (Figure 3B and Supplementary Figure S1). To confirm the binding to these identified sequences, firstly a 59-bp oligonucleotide sequence from −58 to 0 region, upstream of *cprB*-ATG was synthesized. A strongly retarded band of the CprB–DNA complex was observed. The results, as depicted in Figure 3C, show that CprB binds to the 59-bp *cprB*-ATG DNA with a *K*_d_ of ∼400 nM. Two shorter sequences of 27-bp length were subsequently constructed from the 59-bp DNA and it was demonstrated that one of the sequences does not show significant binding to CprB and has very low similarity with the CS. The other 27-bp sequence from −47 to −21 region of the *cprB*-ATG (OPB) exhibits *K*_d_ in the range of 250–300 nM (Figure 3D). A similar trend was observed for the binding of CprB with the *cprA*-ATG upstream sequence from −44 to −20 (Figure 3E). Moreover, to determine the appropriate length of the sequence sufficient for effective interaction of CprB with DNA, both shorter and longer sequences of DNA were synthesized (Supplementary Figure S1a and b). It was concluded that a longer fragment of DNA is not necessary for enhancement of the binding ability of CprB towards DNA. Nevertheless, it was envisioned that these longer sequences of DNA might favor nucleation for effective crystallization of the complex. A detailed list of all DNA sequences analyzed is presented in Supplementary Table S1. Affinity of CprB with the promoters of *scbR* (Figure 4A) and the cryptic type I polyketide gene cluster, *kasO* [*kasO*_B_ (Figure 4B) and *kasO*_A_ (Figure 4C)], both of which are regulated by ScbR, were also tested. Results reveal that CprB binds to these sequences with *K*_d_ ranging from 0.75 to 1.5 μM, which is around three-fold lower than that observed for the OPB sequence. These results indicate that CprB binds to its own upstream sequence with much greater affinity than the *scbR* and *kasO* promoters.

Due to the high sequence similarity in the DBD of CprB with other TetR-FTRs, the binding studies of CprB with their respective target DNA sequences were also tested. The results, as depicted in Supplementary Figure S2A, show that CprB recognizes QacR(OS) at a higher concentration of around 1.5 μM. The binding constant is also approximately five-fold lower than what is exhibited by CprB toward its upstream sequence. Hence, this observation clearly shows that although QacR DNA is recognized by CprB, it is not the preferred choice. A similar set of experiments were also performed with the TetR(OS). However, the 15-mer TetR(OS) did not show any affinity toward CprB (Supplementary Figure S2B). This may be attributed to the inability of CprB to bind with such a short fragment. It is also possible due to the high degree of divergence in the TetR(OS) and CS, CprB does not recognize it. Even at the amino acid sequence level, the HTH motifs of TetR and CprB are divergent; hence, the above observation is not surprising (Supplementary Figure S3).

An attempt to identify the GBL recognized by CprB was performed by employing EMSA with *S. coelicolor* A3(2) extract and a representative GBL molecule (SCB2). Increasing amounts of GBL molecule, SCB2, which is the cognate ligand of ScbR, were added into the reaction mixture of CprB–CS complex. The result shows that SCB2 was unable to break the CprB–CS complex (Supplementary Figure S4A). To further verify if a molecule that could break the CprB–CS complex is present in the extracellular media, we followed the reported protocol to extract GBLs from the liquid culture of *S. coelicolor* A3(2) (58). The prepared extract was able to break the CprB–CS complex (Supplementary Figure S4B), thereby suggesting that the cognate GBL molecule for CprB exists whose identity needs to be determined. This reinforces the idea that CprB, like other GBL receptors, is specific toward its inducers and does not trigger transcriptional activation via binding to generic GBL molecules.

### Deriving thermodynamic binding parameters for CprB–DNA (CS/OPB) complex

**Figure 5.**
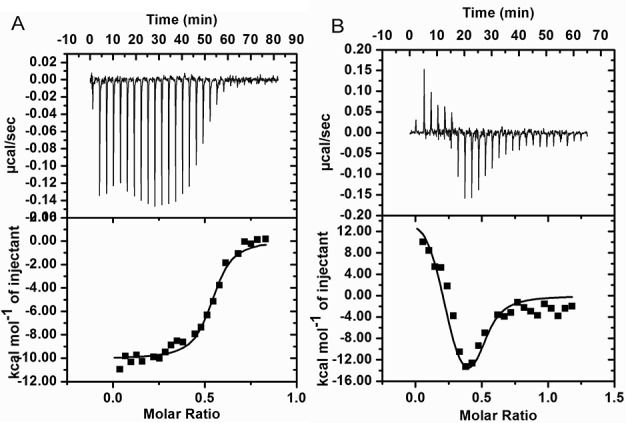
Thermodynamic binding parameters of CprB–DNA were measured using ITC. Raw data are shown in top panel and curve fit in the bottom panel of (**A**) and (**B**). (A) CprB in the sample cell was titrated with 22-mer annealed CS. Data were fit using one set of sites model and the parameters obtained from the curve fitting are as follows: ΔS = –2.7 cal/mol/deg, ΔH = –10.1 kcal/mol, *K*_d_ = 200 nM, n = 0.52, where n is the stoichiometry of bound CS per CprB dimer. (B) CprB titrated against annealed OPB and data were fit using two sets of sites model and the parameters obtained from the curve fitting are as follows: ΔS_1_ = –56.9 cal/mol/deg, ΔH_1_ = –25.8 ± 9.3 kcal/mol, *K*_d_ = 330 nM, n = 0.26 and ΔS_2_ = 86.2 cal/mol/deg, ΔH_2_ = 15.5 ± 1.2 kcal/mol, *K*_d_ = 33 nM, n = 0.23.

The thermodynamic parameters for the formation of the CprB–CS and the CprB–OPB complexes were determined by performing ITC experiments. As depicted in Figure 5A, the titration of 20 μM CprB to 120 μM of CS shows a complex formation with an exothermic heat of binding. As the complex formation progressed, an incremental heat release was observed as shown in the lower panel of the binding isotherm (Figure 5A). Using an equilibrium-binding model, the data acquired were fit using one set of sites model and the *K*_d_ as well as other thermodynamic parameters like ΔH, ΔS and ΔG were obtained. The enthalpy and the entropy values for the formation of CprB–CS complex are ΔH = –10.1 kcal/mol, ΔS = –2.7 cal/mol/deg, respectively (Figure 5A). The large value of ΔH suggests that the interaction between protein and DNA is highly enthalpy driven. The formation of non-covalent interactions between the two interacting molecules is assumed to be the major contribution toward the affinity. The *K*_d_ of CprB with CS is 200 nM and the change in Gibbs free energy for this reaction is ΔG = –9.27 kcal/mol. In contrast to CprB–CS complex, the thermodynamic data obtained for CprB–OPB interaction were fitted using two sets of sites model with *K*_d__1_ = 330 nM and *K*_d__2_ = 33 nM (Figure 5B). The binding model consistent with this observation is supported by an initial binding of one of the CprB dimers to the OPB sequence, which is most likely an enthalpy-driven process. This is followed by the binding of the second dimeric unit of CprB that is most likely an entropy-driven step (Figure 5B). The values of the *K*_d_ in both cases, CS and the OPB sequence, are in agreement with the results of the DNA retardation assays. In the latter case, since the particulars of the binding mechanism cannot be observed, the values obtained by EMSA are closer to an average of the two binding constants calculated via ITC.

### Mutagenesis in the DBD of CprB

**Figure 6.**
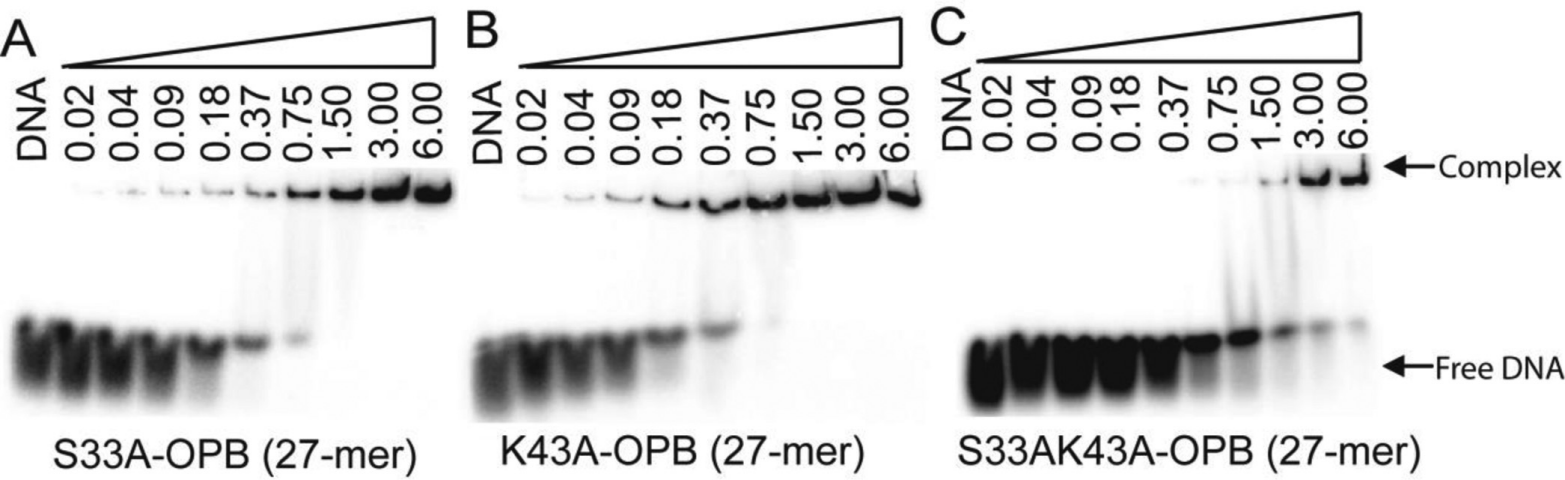
EMSA studies of mutant CprB carried out with *cprB* upstream sequence (OPB). (**A**) Backbone interacting residue S33 was mutated to alanine residue in native CprB; (**B**) base interacting residue K43 was mutated to alanine residue; and (**C**) double mutation of S33 and K43 residues to alanine residue. Single mutations have negligible effect on DNA binding. Double mutants, however, result in drastic decrease in DNA binding ability. All the concentrations are mentioned in micromolar.

To decipher the importance of the structural basis of interaction of CprB with the DNA, a couple of variants of CprB were designed via inspection of the CprB–CS complex crystal structure. First, a series of single amino acid mutations in CprB were made. All residues selected for mutagenesis are from the spacer helix α2 and recognition helix α3. Representative mutations disrupting the phosphate backbone interactions constructed were S33A and T31A and the base interacting single mutants engineered were Y47A, F48A and K43A. The mutant proteins were purified and tested for binding with both the OPB and the *cprA*-ATG upstream sequences. The results of the single point mutation in CprB showed that there is almost no effect on the ability of the protein to bind with DNA (Figure 6A and B). This highlights the importance of the fact that single mutations can be absorbed by this class of proteins without affecting DNA binding ability of CprB. Further, to investigate the ability of the CprB to absorb multiple mutations, a double mutation (S33A, K43A) which disrupts both a phosphate backbone contact and a DNA base interaction was constructed. The double mutant showed a pronounced effect (Figure 6C). We conclude that since the DNA makes several interactions with the four monomers of the protein forming an extensive interface, disruption of a few interactions does not affect DNA binding. On the other hand, the creation of double mutant results in a cumulative reduction of protein–DNA contacts, thereby drastically impairing the DNA binding capability of the protein.

## DISCUSSION

### Comparison of TetR-FTRs

Most of the TetR-FTR proteins are involved in antibiotic production and resistance pathways, where they serve as regulators by binding to diverse small molecule effectors (25). An analysis of the seven protein–DNA complex structures available from the TetR-FTRs reveals that they have evolved into two different sub-classes. One of them binds to their cognate DNA element as a dimer and the other binds as dimer of dimers. For example, *S. aureus* QacR (28), *C. glutamicum* CgmR (30), *E. coli* SlmA (32) and *M. smegmatis* MS6564 (33) bind their cognate DNA as dimer of dimers. Whereas proteins like *E. coli* TetR (27), *P. aeruginosa* DesT (29) and *S. antibioticus* SimR (31) recognize their DNA as homodimers. There are several differences between these two sub-classes of TetR-FTRs. An analysis shows that the dimer of dimers DNA binding proteins in general (QacR, 28-bp (28), CgmR, 32-bp (30), MS6564, 31-bp (33) and CprB, 22-bp) recognize longer stretch of DNA sequences than the dimeric DNA binding proteins [TetR, 15-bp (27), SimR, 17-bp (31) and DesT,17-bp (29)]. Although both the sub-classes of TetR-FTRs introduce a bend in their DNA to recognize their cognate sequences, the distortion is more significant for the dimeric sub-class. For example, TetR and SimR bend their DNA around 17° and 15°, respectively (27,31), whereas QacR and CprB introduce a bend of only around 3° and 3.5°, respectively (28). This gets reflected in the center-to-center distance of 37 and 38.2 Å observed in QacR and CprB, respectively, as opposed to 31 and 32.2 Å in the cases of TetR and SimR, respectively. While the dimeric DNA binding proteins achieve specificity and high affinity by global bending of the DNA, the latter TetR-FTRs achieve this by increasing the total number of protein–DNA contacts via binding of two dimers to a longer stretch of DNA.

**Figure 7.**
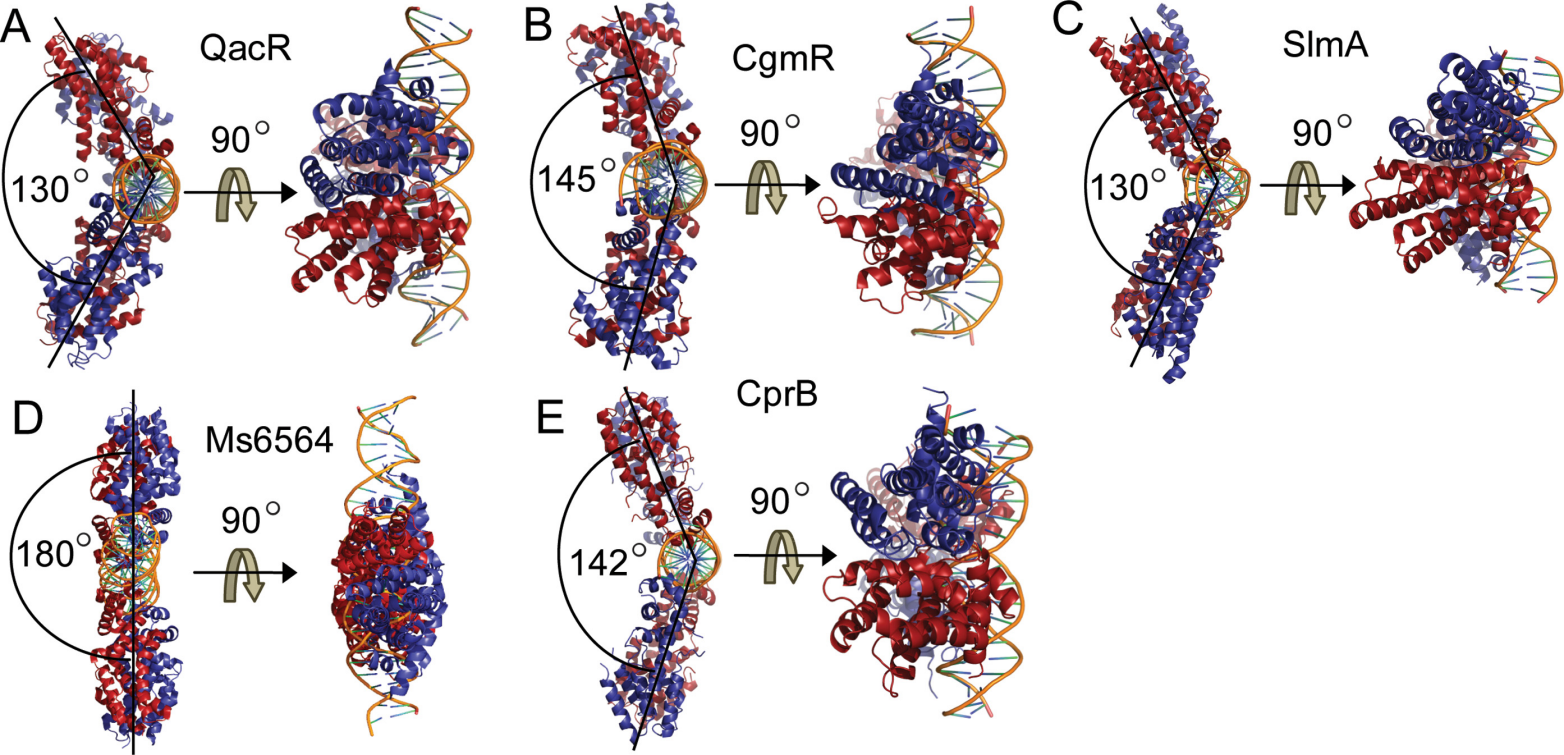
Comparison between the structures of DNA binding proteins from TetR-FTR that form dimer of dimers. The angle between the solid lines passing through the center of the dimer with respect to the DNA is shown in (**A**–**E**). The top view is obtained by rotating the structure by 90° along the *x*-axis as indicated in the figure. The PDB codes used for comparison are 1JT0, 2YVH, 4GCT, 4JL3 and 4PXI for QacR, CgmR, SlmA, Ms6564 and CprB, respectively.

Within the dimer of dimers sub-class, even though the mode of binding is similar, an analysis of the structures shows that the angles between the two homodimers with respect to the DNA differs (Figure 7). The angle between the homodimers of CprB and DNA is found to be 142°. For the QacR–DNA (Figure 7A), CgmR–DNA (Figure 7B) and SlmA–DNA complexes (Figure 7C), it is 130°, 145° (30) and 130°, respectively. In the case of MS6564–DNA complex, this bend is not present and the angle is around 180° corresponding to a conformation closer to the ideal B-form (shown in Figure 7D). It appears that the angle between the homodimers is an alternative representation of the distortion introduced into the DNA upon protein binding and can be broadly correlated to the specificity of DNA recognition. For instance, CgmR, QacR, SlmA and CprB that introduce distortion in the DNA, are sequence-specific regulators. They identify their cognate DNA sequences by an induced-fit mechanism through widening of the groove to enable requisite protein–DNA contacts. In contrast, MS6564, a global regulator does not induce a deformation in the DNA structure and interacts with the DNA through a network of water molecules, ensuring proper scanning over a long stretch of generic sequences (33).

Another difference between these two sub-classes is that in several instances, the dimer of dimers TetR-FTRs recognize a non-palindromic sequence. As a consequence of this heterogeneity in the DNA sequence, each of the four HTH motifs makes contacts with regions in DNA that are non-analogous. This exposes the pair of dimers to different environments making this sub-class more tolerant than the dimeric TetR-FTRs in accepting varied DNA sequences. Further, the ITC results of both QacR (28,59) and CprB demonstrate that they both exhibit a cooperative mode of binding to their respective OS. The cooperativity of DNA binding is most likely conferred by the non-palindromic nature of the DNA sequences recognized by the dimer of dimers. The ITC results of the CprB with its OPB show two-state binding, in which the first binding constant is 10 times weaker than that of second binding constant. We propose that the binding of CprB to DNA occurs via a clamp and click mechanism. In the first step, both the dimeric units interact via one of the monomers with the DNA in an enthalpy-favored process (Figure 5B). This binding event induces a conformational twist in the DNA, priming it for the second step that results in clicking the other two monomeric units of each dimer present on either side of the DNA. The second step is, therefore, entropy driven (Figure 5B) and plausibly occurs by pushing out the water molecules from the major groove of the DNA during the formation of the protein–DNA complex.

Even at the ligand-binding level, a comparison of the two types of TetR-FTRs shows that almost all proteins in the dimeric sub-class bind to very specific effector molecules. For example, TetR is specific to tetracycline and utilizes co-inducer molecules like Mg^2+^ to alter its operator-binding form (27,34,35,37,38). Moreover, apart from the usual four helix bundle (α6–α9 in CprB) that forms the wall of the inducer pocket, the dimeric sub-class additionally possesses helical insertion motifs between α8 and α9 that interact with the ligand and confer specificity (27,31). On the other hand, the TetR-FTRs that bind to DNA as a pair of dimers do not possess these insertions and instead have more accessible binding pockets (28,30) that can accommodate a broad spectrum of signaling molecules. For instance, QacR accepts a host of quaternary ammonium cationic moieties and the binding of the bulky inducers results in deformation of the ligand-binding pocket that triggers transcriptional activity (40). Similarly, CprB seems to possess a large cavity that can potentially accept a variety of ligands.

### Model of DNA binding

**Figure 8.**
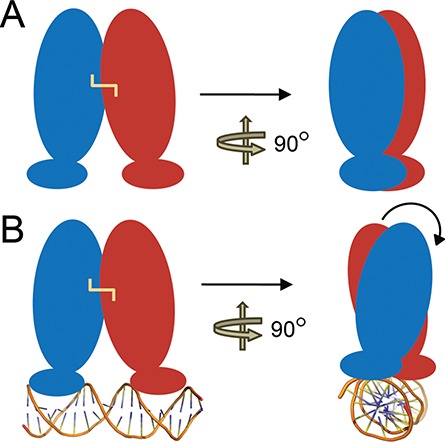
A model representing the changes induced upon DNA binding in the TetR-FTR protein, CprB. Two forms of protein are shown in the figure, apo form (**A**), and operator (DNA) bound form (**B**). The operator binding induces a twist in the dimeric interface of the homodimer and facilitates the snug fit of HTH motif in the major groove of the DNA. Intermonomeric disulfide bond is represented as yellow line and the DNA in cartoon model.

To map the various conformational states that the TetR-FTR class of transcription factors access, a comparison of the apo, ligand and DNA-bound forms of the available structures was performed. Analyses showed that a global twist along the dimeric motif occurs in each case upon DNA binding. This results in restructuring of several interactions across various regions of the protein with the effect being most pronounced at the dimeric interface. As reported here, for CprB, ∼10 hydrogen bonds were restructured (Supplementary Tables S2 and S3). Our analyses show that a similar scenario is also observed for other members like QacR (PDB entry: 1JT0), CgmR (PDB entry: 2YVH) and SimR (PDB entry: 3ZQL) proteins, where DNA binding induces a considerable rearrangement of the specific interactions and many new hydrogen bonds were formed across the interface in each case. In all these proteins, a concurrent coordinated shift that facilitates the insertion of the recognition helix into the major groove of the DNA is also observed (similar to that shown for CprB, Figure 2A). This overall motion in the structures upon DNA binding results in locking the protein in a conformationally favorable state toward accepting inducers by increasing the accessibility to the ligand-binding pocket. Hence, it can be concluded that conformational changes are facilitated through a pendulum-like rearrangement of the fluid dimeric interface (Figure 8). Each monomer rotates along the dimeric axis with the direction of rotation being determined so as to facilitate operator binding. These kinds of allosteric transitions that facilitate function are seen in various transcription factors that are regulated by small molecule inducers (25,60). For example, the well-established system, LacR, similar to TetR-FTRs, also adopts different conformations in the DNA, and the small molecule repressor (IPTG) bound forms; a subtle switch between these states controls its transcriptional activity (61). In contrast, there are also systems like the tetrameric gene regulator, TtgV, a multidrug-binding protein and controller of efflux pump, which undergo drastic conformational changes (like the wings of a flying bird) in structure between its operator and inducer-bound forms to assist function (62).

### Insights into the functions of CprB

A genome-wide search for the cognate DNA of CprB in *S. coelicolor* A3(2) followed by EMSA experiments was performed to identify the cognate DNA element regulated by CprB. The results revealed that CprB is a potential autoregulator of its own gene and also likely regulates expression of the *cprA* gene. This was not a surprise finding as many of the well characterized TetR-FTRs not only serve as antibiotic production modulators or efflux regulators, but are also autoregulatory in nature. For example, *S. coelicolor* A3(2) proteins, ScbR and ActR regulate their own production by binding to the intergenic sequence of *scbR-scbA* (20) and *actR*-*actA* (63), respectively, and additionally regulate other antibiotic pathways in this species. Similarly, *sco*3201 (64), PhoP (65) and HspR (66) from *S. coelicolor* A3(2) are also TetR-FTRs that act as autoregulatory proteins. Apart from *S. coelicolor* A3(2), other *Streptomyces* strains like *S. fradiae* also host TetR-FTRs that operate as autoregulators. For instance, TylP, regulates its own expression and also controls tylosin biosynthesis in *S. fradiae* (16,67). Other TetR-FTRs like *S. clavuligerus* putative regulatory protein CcaR (68), *E. coli* efflux protein TetR (36,69) and *Pseudomonas putida* regulatory protein PsrA (70) also displayed autoregulatory properties.

Our studies showed that apart from its own upstream sequence, which it binds with much greater affinity, CprB recognizes the cognate sequences for ScbR (promoter sequences of *scbR* and *kasO*) with affinity in low micromolar range. This information is in accord with the very recent discovery of the fourth GBL receptor of TetR-FTRs, ScbR2 from *S. coelicolor* A3(2). ScbR2 was identified as a pseudo GBL receptor and it was shown that it binds to the *kasO* and *scbA* promoter sequences, and regulates the production of ScbA (24). It was further demonstrated that instead of utilizing GBL molecules as trigger, ScbR2 gets activated by endogenously produced antibiotics like undecylprodigiosin and actinorhodin (18,24). Hence, in *Streptomyces*, antibiotic regulation is most likely multilayered involving many regulatory proteins. It appears that in these organisms, there are plethora of regulatory systems that communicate the general state of the species like environmental stress, population density and other secondary metabolic pathways. In addition, there are also cluster-specific regulators that reside within the antibiotic biosynthesis gene cluster and control their production. The cross-talk between these pathway specific and pleiotropic regulators probably influences the onset of antibiotic production. This can happen either by binding of antibiotic or their intermediates to these regulatory protein systems and/or via interaction with specialized signaling molecules like γ-butyolactones, thereby resulting in a self-reinforcing feed-forward circuitry (71).

A plausible model of regulation via the GBL receptor sub-class of proteins in *S. coelicolor* A3(2) could be envisioned as ScbR being the principal GBL-binding protein and the other three proteins, CprA, CprB and ScbR2 as indirect positive or negative regulators of the pathway ScbR controls. A similar scenario is observed in the *S. fradiae* system where a host of putative GBL-dependent regulators like TylP, TylQ, TylS, TylU and TylR are known to modulate tylosin production through their coordinated expression. In this system, TylP is at the top of the regulatory network and the rest of the putative GBL receptors assist in controlling tylosin production via an intricate network of positive and negative feedback mechanism (16,67). Therefore, it is likely even in *S. coelicolor* A3(2), a fine interplay of GBL receptors is present that controls the secondary metabolic pathways in this species. However, the details of how each member of this network controls self-expression levels as well as the function of the other individual members remains elusive and hence there is need for further study.

## SUPPLEMENTARY DATA

Supplementary data are available at NAR Online.

SUPPLEMENTARY DATA
