# Supplementary material for: Structural and functional basis of transcriptional regulation by TetR family protein CprB from S. coelicolor A3(2)
Source: Nucleic Acids Res. 2014 Aug 4;42(15):10122–33. doi: 10.1093/nar/gku587 (PMC4150764; doi:10.1093/nar/gku587)
Supplement: SUPPLEMENTARY DATA [file supp_42_15_10122__index.html]

Structural and functional basis of transcriptional regulation by TetR family protein CprB from S. coelicolor A3(2) — Structural and functional basis of transcriptional regulation by TetR family protein CprB from S. coelicolor A3(2) — SUPPLEMENTARY DATA 

# Structural and functional basis of transcriptional regulation by TetR family protein CprB from *S. coelicolor* A3(2)

## SUPPLEMENTARY DATA

**Files in this Data Supplement:**

- SUPPLEMENTARY DATA
