# Supplementary material for: Structural and functional basis of transcriptional regulation by TetR family protein CprB from S. coelicolor A3(2)
Source: Nucleic Acids Res. 2014 Aug 4;42(15):10122–33. doi: 10.1093/nar/gku587 (PMC4150764; doi:10.1093/nar/gku587)
Supplement: SUPPLEMENTARY DATA [file supp_gku587_nar-00818-h-2014-File003.doc]

**Supporting information**

Table S1: List of oligonucleotides synthesized for EMSA studies

| S.No. | Name | Sequence (5' to 3') |
| --- | --- | --- |
| 1  2  3  4  5  6  7  8  9  10  11  12  13  14  15  16  17  18  19  20  21  22 | CS[1]  CS[2]  PCS_1_  PCS_2_[1]  PCS_2_[2]  TetR(OS)[1]  TetR(OS)[2]  QacR(OS)  scbR[1]  scbR[2]  *kasO*_A_[1]  *kasO*_A_[2]  *kasO*_B_[1]  *kasO*_B_[2]  *cprB*_1_[1]  *cprB*_1_[2]  *cprB*_2_[1]  *cprB*_2_[2]  *cprA*_1_[1]  *cprA*_1_[2]  *cprA*_2_[1]  *cprA*_2_[2] | ACATACGGGACGCCCCGTTTAT  ATAAACGGGGCGTCCCGTATGT  CTTATATACGGGACGTCCCGTATATAAG  CGCGACATACGGGACGTCCCGTATATGAGG  CCTCATATACGGGACGTCCCGTATGTCGCG  TCTATCATTGATAGG  CCTATCAATGATAGA  CTTATAGACCGATCGATCGGTCTATAAG  GGAACCGGCAATGCGGTTTGTTCGATC  GATCGAACAAACCGCATTGCCGGTTCC  ACAAACCGGTGTGCTGGTTTGTAAAGTCGTGG  CCACGACTTTACAAACCAGCACACCGGTTTGT  CAAAACAGACTTGTTAGCTGTTT  AAACAGCTAACAAGTCTGTTTTG  GCCGGGGGTAAAGGCAGGCGGCACGGTCTGTT  GAGTTCCAGCCCGGGAGAATCCAGCCG  CGGCTGGATTCTCCCGGGCTGGAACTCAACAG  ACCGTGCCGCCTGCCTTTACCCCCGGC  AGGCAGGCGGCACGGTCTGTTGAGTTC  GAACTCAACAGACCGTGCCGCCTGCCT  AACAGGCACACGGTCTGTTGGTTCT  AGAACCAACAGACCGTGTGCCTGTT  TCTCAGCCCGGGAGAGTCCAGCCGA  TCGGCTGGACTCTCCCGGGCTGAGA |

Labels of the oligonucleotides synthesized. Consensus sequence (CS). PCS_1_ and PCS_2_ are perfectly palindromic sequences of CS with chain lengths 28-mer and 30-mer respectively. TetR(OS) and QacR(OS) are the operator sequences (OS) of TetR and QacR respectively. ScbR recognition sequences are *scbR*, *kasO*_A_ and *KasO*_B_. *cprB*_1_ and *cprB*_2_ are the upstream (−58 to 0 and −47 to −21 respectively) sequences of *cprB*-ATG. *cprA*_1_ and *cprA*_2_ are the upstream (−44 to −20 and −23 to 1) of *cprA*-ATG respectively. [1] and [2] in each sequence listed above in the table are complementary to each other.

Table S2: List of hydrogen bonding interactions disrupted upon DNA binding

| Chain A | | Chain B | | Distances Å | |
| --- | --- | --- | --- | --- | --- |
| Residue | Atom interacting | Residue | Atom interacting | Distance in apo form | Distance in DNA bound form |
| 28 Glu | OE1(O) | 28 Glu | OE1(O) | 3.1 | 6.2 |
| 111Thr | O(O) | 108Arg | NH2(N) | 2.9 | 4.0 |
| 145His | NE2(N) | 190Arg | NE(N) | 3.3 | 8.6 |
| 145His | NE2(N) | 190Arg | NH2(N) | 3.0 | 9.1 |
| 149Asp | N(N) | 183Glu | OE2(O) | 2.8 | 8.0 |
| 149Asp | OD2(O) | 179Arg | NH1(N) | 3.3 | 6.3 |
| 155His | NE2(N) | 172Glu | OE1(O) | 3.4 | 6.8 |
| 155His | NE2(N) | 164Thr | OG1(O) | 3.4 | 4.0 |
| 190Arg | NE(N) | 191Gly | O(O) | 2.9 | 3.7 |
| 190Arg | NH2(N) | 191Gly | O(O) | 3.1 | 4.6 |

Atom in the parenthesis are involved in the interaction. O(O) and N(N) are the atoms from the peptide backbone and rest are from the side chain atoms of the protein.

Table S3: List of hydrogen bonding interaction formed in the DNA bound form of CprB

| **Chain C** | | **Chain D** | |  |
| --- | --- | --- | --- | --- |
| Residue | Atom interacting | Residue | Atom interacting | Distance Å |
| 29Ser | OG(O) | 28Glu | OE1(O) | 3.3 |
| 108Arg | NH2(N) | 113Gly | N(N) | 2.8 |
| 112Ala | O(O) | 108Arg | NH2(N) | 3.3 |
| 152Cys | OG(S) | 179Arg | NH1(N) | 3.2 |
| 155His | O(O) | 163Gly | O(O) | 3.0 |
| 155His | ND1(N) | 180Arg | NH2(N) | 3.1 |
| 159Cysr | N(N) | 163Gly | O(O) | 2.9 |
| 180Arg | NE(N) | 155His | ND1(N) | 3.1 |
| 180Arg | NH2(N) | 155His | ND1(N) | 2.9 |
| 191Gly | O(O) | 190Arg | NH1(N) | 3.4 |

Atom in the parenthesis is involved in the interaction. O(O) and N(N) are the atoms from the peptide backbone and rest are from the side chain atoms of the protein.

Table S4: Local base-pair parameters

| S.No. | Step | Shift | Slide | Rise | Tilt | Roll | Twist |
| --- | --- | --- | --- | --- | --- | --- | --- |
| 1  2  3  4  5  6  7  8  9  10  11  12  13  14  15  16  17  18 | AT/AT  TA/TA  AC/GT  CG/CG  GG/CC  GG/CC  GA/TC  AC/GT  CG/CG  GC/GC  CC/GG  CC/GG  CC/GG  CG/CG  GT/AC  TT/AA  TT/AA  TA/TA | -0.66  0.56  0.53  1.23  -1.25  0.17  0.18  0.61  -1.48  -0.19  0.96  0.64  -0.59  -0.25  0.57  -0.30  -1.11  -0.07 | -2.62  0.13  -0.46  0.19  -0.95  0.57  0.19  0.08  0.17  -0.14  -0.13  -0.04  0.09  1.31  -2.23  -0.13  -0.14  0.27 | 3.61  3.76  3.40  3.31  3.50  3.32  3.73  3.14  3.52  3.48  3.31  3.51  3.80  3.31  3.32  3.49  3.31  3.77 | -4.88  -2.23  -1.22  -3.14  2.91  3.04  3.59  1.77  -7.08  -0.95  8.92  -2.99  -4.36  -1.54  1.01  1.65  3.29  -1.42 | -10.35  2.89  -4.84  -6.84  1.60  5.41  -7.06  8.77  0.78  2.02  -5.32  10.90  -5.87  2.16  -0.04  -2.86  -3.98  2.27 | 26.55  47.17  40.84  37.94  33.35  26.64  33.55  29.55  32.93  37.27  27.74  31.47  35.79  35.98  21.04  34.33  44.48  53.79 |
|  | ave.  s.d. | -0.03  0.77 | -0.21  0.92 | 3.48  0.19 | -0.20  3.84 | -0.58  5.73 | 35.02  7.98 |

DNA base-pair parameters obtained by using 3DNA program. Average and standard deviations are denoted as ave. and s.d. respectively.

Figure S1


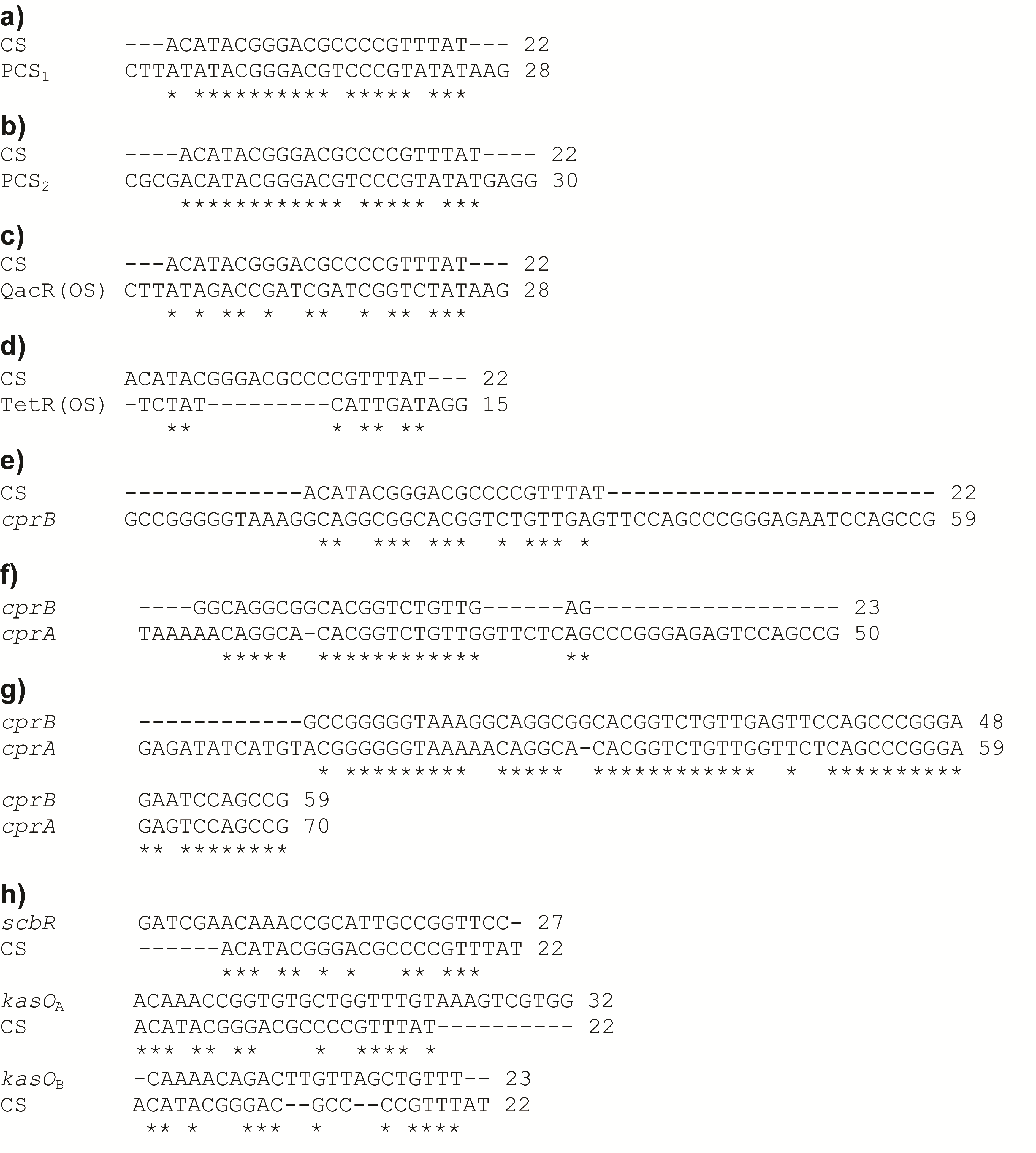


Figure S1. ClustalW alignment of DNA sequences showing the similarity with the CS. a), b) A longer stretch (28-mer and 30-mer) of CS with mutations in the bases to attain perfect palindromic sequence (PCS_1_ and PCS_2_). c) and d) QacR(OS) and TetR(OS) operator sequences were aligned to CS. e) Alignment of CS with 59-mer (−58 to 0) of *cprB*-ATG upstream sequence. f) and g) Comparison in the upstream sequences of *cprA*-ATG (−69 to 0) and *cprB*-ATG (−58 to 0) genes. The longer sequence of *cprA*-ATG upstream sequence (70-mer) was taken to see where exactly the 59-mer of *cprB*-ATG upstream sequence aligns to. h) ScbR binding sequences (*scbR, kasO*_A_ and *kasO*_B_) were aligned to CS.

Figure S2


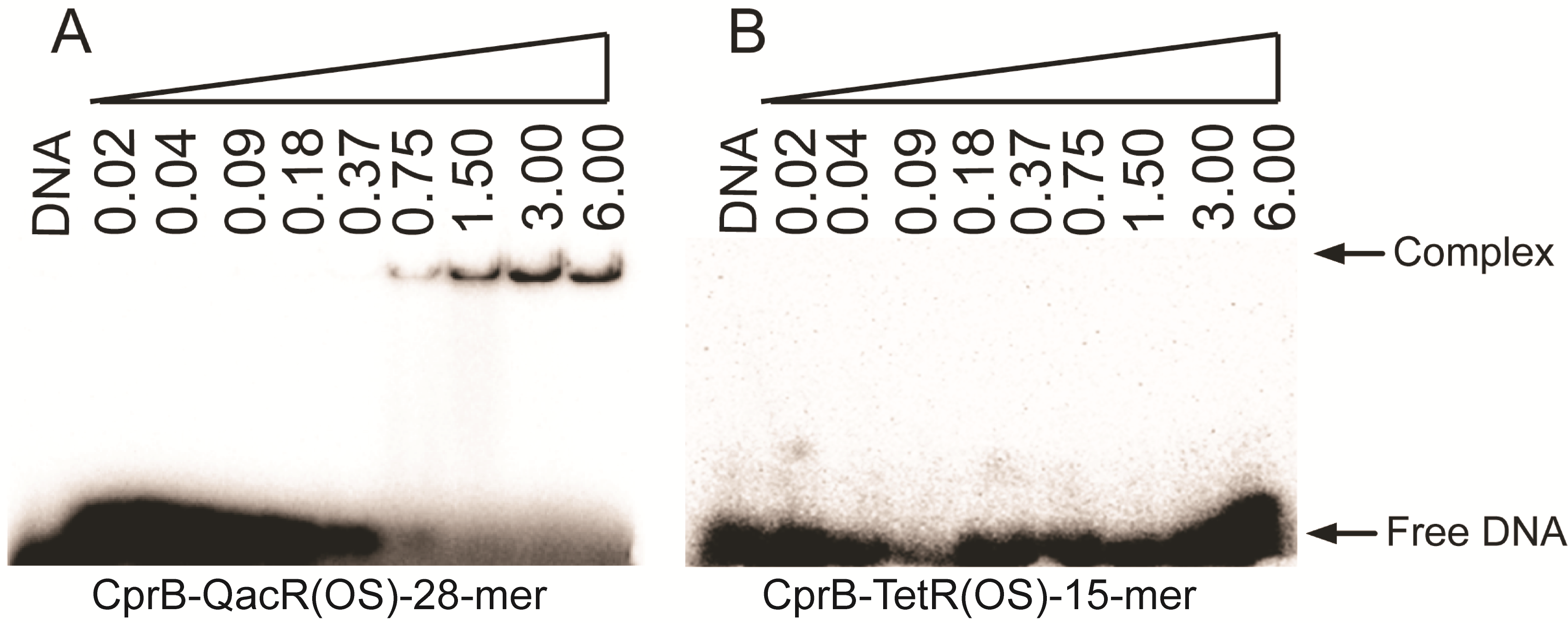


Figure S2. Results of DNA retardation assay performed with CprB and A) the QacR (28-mer) and B) TetR (15-mer) operator sequences (OS).

Figure S3


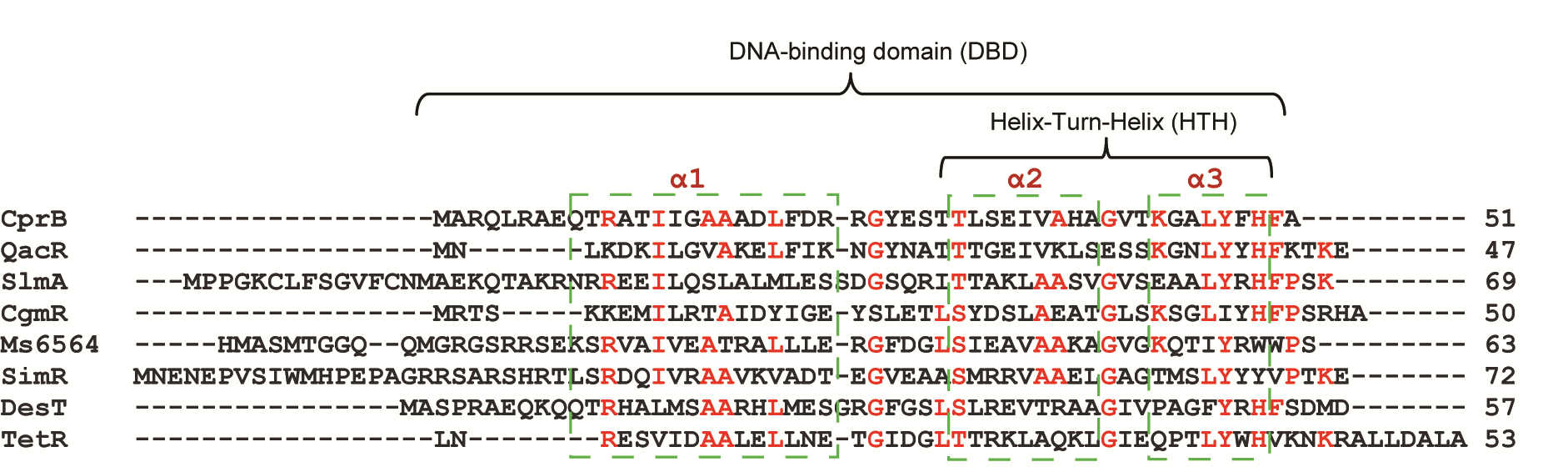


Figure S3. The alignment of amino acids (aa) in the N-terminal DBDs of the proteins in TetR-FTRs whose structures are reported till date. The helices α1-α3 is given in green dashed line boxes. The aa highlighted in red are conserved in four or more proteins across the alignment. The aa comprising DBD and the HTH-motif are labeled. CprB shows more aa sequence similarity to QacR, characteristically in the recognition helix (α3).

Figure S4


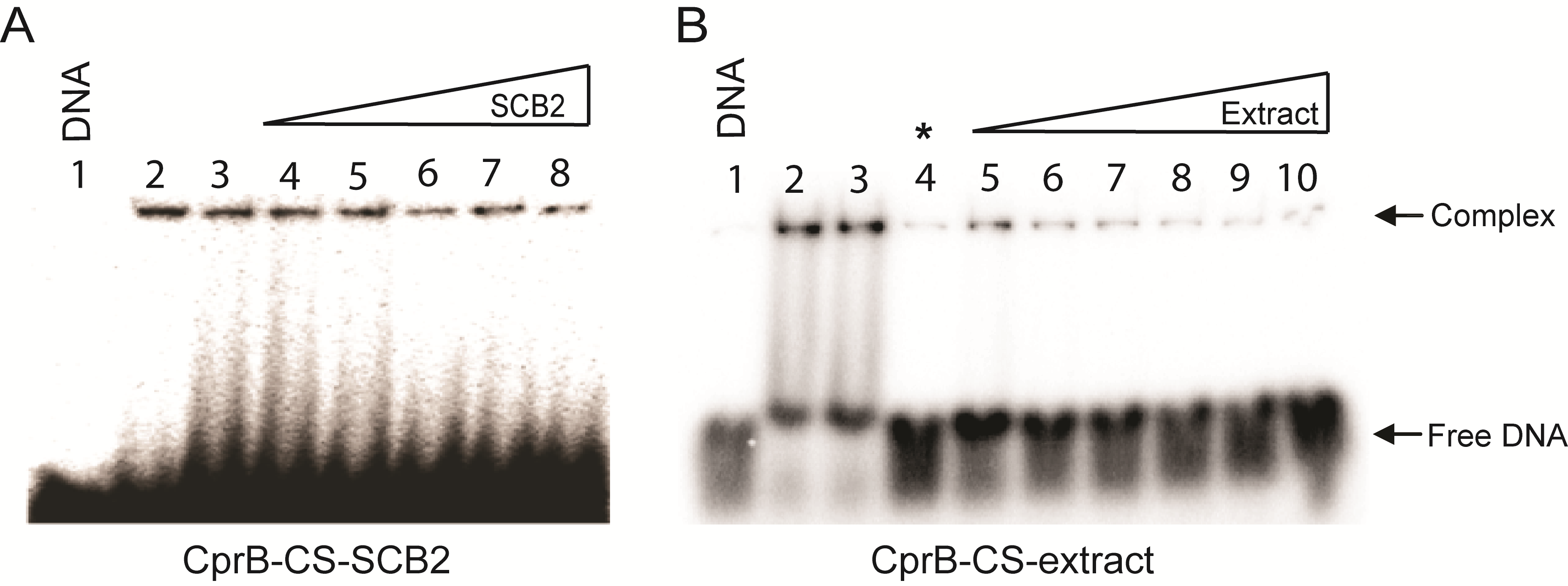


Figure S4. CprB-CS complex breaking assays. A) Chemically synthesized SCB2 was used to break CprB-CS complex. Amount of protein added was 500 nM in all lanes except Lane1 which has only CS. Lane2 and 3 are set as control, in which Lane2 has only protein and CS in the reaction mixture, whereas in Lane3, it has protein, CS and 3% DMSO (Dimethyl sulfoxide) in reaction mixture. Lanes4 to 8 has increasing amounts (100 nM to 500 nM ) of SCB2 dissolved in 3% DMSO. B) Concentrated extract from the liquid culture of *S*. *coelicolor* A3(2) was used to study the CprB-CS complex breaking. Lane1 has only CS and in all lanes the protein concentration used was 500 nM. Lane3 has protein-DNA complex in 5% methanol. Lane4 labeled with asterisk has excess of cold CS along with the reaction mixture. Lane5 to Lane10 increased amount of extract (dissolved in 5% methanol) added to the CprB-CS complex.
